# Body Mass, Physical Activity and Eating Habits Changes during the First COVID-19 Pandemic Lockdown in Poland

**DOI:** 10.3390/ijerph18115682

**Published:** 2021-05-26

**Authors:** Hubert Dobrowolski, Dariusz Włodarek

**Affiliations:** 1 Faculty of Medical Sciences and Health Sciences, University of Social and Medical Sciences in Warsaw, 04-367 Warsaw, Poland; 2 Department of Dietetics, Institute of Human Nutrition Sciences, Warsaw University of Life Sciences (SGGW), 02-787 Warsaw, Poland; dariusz_wlodarek@sggw.edu.pl

**Keywords:** COVID-19, lockdown, physical activity, eating habits, body mass changes

## Abstract

The outbreak of the COVID-19 pandemic caused a number of changes in social life around the world. In response to the growing number of infections, some countries have introduced restrictions that may have resulted in the change of the lifestyle. The aim of our study was to investigate the impact of the lockdown on body weight, physical activity and some eating habits of the society. The survey involving 183 people was conducted using a proprietary questionnaire. The mean age of the study participants was 33 ± 11 and mean height 169 ± 8 cm. An average increase in body weight was observed in 49.18% by 0.63 ± 3.7 kg which was the result of a decrease in physical activity and an increase in food consumption. We also observed a decrease in PAL from 1.64 ± 0.15 to 1.58 ± 0.13 and changes in the amount of food and individual groups of products consumption, including alcohol. Among the study participants who did not lose body mass, there was an average weight gain of 2.25 ± 2.5 kg. In conclusion, an increase of weight was shown in about half of the respondents in the study group which was associated with a decrease in physical activity and an increase in the consumption of total food and high energy density products.

## 1. Introduction

At the end of 2019, a new type of coronavirus (CoV) was detected in the Wuhan area of Hubei province in China which led to hospitalization of the first patients [1] causing the development of respiratory symptoms and its failure, similar to the previously discovered CoV inducing severe acute respiratory syndrome (SARS) [2]. A new virus (SARS-CoV-2) led to the development of a new disease (COVID-19) most often causing a fever, dry cough, breathing difficulties (dyspnea), headache and pneumonia, and may result in progressive respiratory failure due to the damage of alveoli, and even death [2,3].

Over the course of several months, SARS-CoV-2 has spread to most countries causing a global pandemic. In Europe, the first cases were diagnosed in Italy and Spain, where the virus wreaked the greatest havoc, causing about 130,000 infections and over 15,000 and 12,000 deaths, respectively, in just over a month [4]. Based on the experience of these countries, other ones have constructed their own way of fighting CoV, from more liberal (like the Swedish model) to more restrictive (e.g., India) methods. In Poland, despite the initial small number of reported cases of COVID-19, a decision was made to introduce a lockdown from mid-March 2020, limiting the mobility of citizens to the necessary activities in everyday life (commuting to work, necessary grocery shopping), closing the vast majority of retail and service outlets, significantly reducing the number of people allowed to stay in one room (e.g., in shops or public transport), ordering the mouth and nose to be covered in public spaces, or finally prohibiting access to parks and forests.

Without judging the economic consequences, the total lockdown lasting about a month meant that Poland significantly reduced the number of new infections and deaths in the initial phase of the pandemic and the sharp increase in infections only started in October, 2020. However, the confinement of society at home has caused drastic changes in the way citizens live. With few exceptions, job closures and a ban on leaving the home have certainly limited mobility which was also the assumption of the restrictions. The first national studies identified psychological problems as a result of lockdown in various social groups [5,6,7,8]. Changes in eating behavior were also observed [9,10,11,12].

A higher risk of severe COVID-19 has been observed in patients with established comorbidities, often dependent on diet [13,14]. Given the reduced mobility and thus daily physical activity, eating behavior changes, as well as possible excessive or deficient food consumption caused by psychological stress, it may be suspected that there may be significant changes in body weight in the society during government restrictions caused by the pandemic. These changes may, however, result in weight gain leading to the development of overweight and obesity and, consequently, to an increased risk of developing diseases that increase the severity and mortality among those infected with SARS-CoV-2.

Hence, the aim of this study was to check the differences in body weight, physical activity and selected eating habits that may have a detrimental effect on body weight and the development of metabolic diseases during the first lockdown at the turn of the first and second quarter of 2020 in Poland.

## 2. Materials and Methods

The pilot study using the proprietary questionnaire was carried out using the CAWI method (Computer-Assisted Web Interview). A total of 183 people took part in the studies (143 women and 40 men). Most of the respondents lived in large cities (>500,000 inhabitants)—*n* = 102, then village—*n* = 31; and also: cities <10,000 inhabitants, *n* = 10; cities with 10,000–100,000 inhabitants, *n* = 20; and cities with 100,000–500,000 inhabitants, *n* = 19. The questionnaire included pre-lockdown and post-lockdown weight, as well as age and height questions with short instruction about body weight measurement. The respondents were asked for self-assessment which factors, in their opinion, influenced the most the changes in body mass.

Physical activity before the lockdown and when the highest number of government restrictions was imposed was performed using a Johansson and Westerterp questionnaire [15]. This questionnaire is useful method to provide information about physical activity level, and was validated before with double-labeled water method before [15]. Therefore, the people taking part in the study were to assess their professional and non-occupational physical activity before and during the lockdown. Based on these data, the physical activity level (PAL) at these two moments was determined. In addition, the question was asked whether the number of heavy household duties (scrubbing floors, cleaning windows, etc.) increased while being locked in the house.

Later in the questionnaire, the respondents were asked to define changes in consumption according to the five-point Likert scale (significantly decreased, slightly decreased, did not change, slightly increased, significantly increased) total food; sweetened and confectionery products; salty snacks, fast-food and sweetened beverages; alcohol; and take-away/home delivery.

Basal metabolic rate (BMR) was calculated using the Harris–Benedict formula, on the basis of body weight, height, sex and age for the pre-lockdown period, as well as in its culminating part. Total energy expenditure (TEE) was calculated by multiplying the BMR by the PAL estimated from the Johansson and Westerterp questionnaire.

Statistical analysis was conducted by means of the SPSS v. 20 software (IBM Corp., Armonk, NY, USA). To verify the normality of distribution, the Shapiro–Wilk test was used. To compare obtained results between different groups the Wilcoxon test and Kruskal–Wallis test were applied. The correlation was based on Spearman’s test. The study’s defined significance level was set to α = 0.05.

## 3. Results

**Figure 1:**
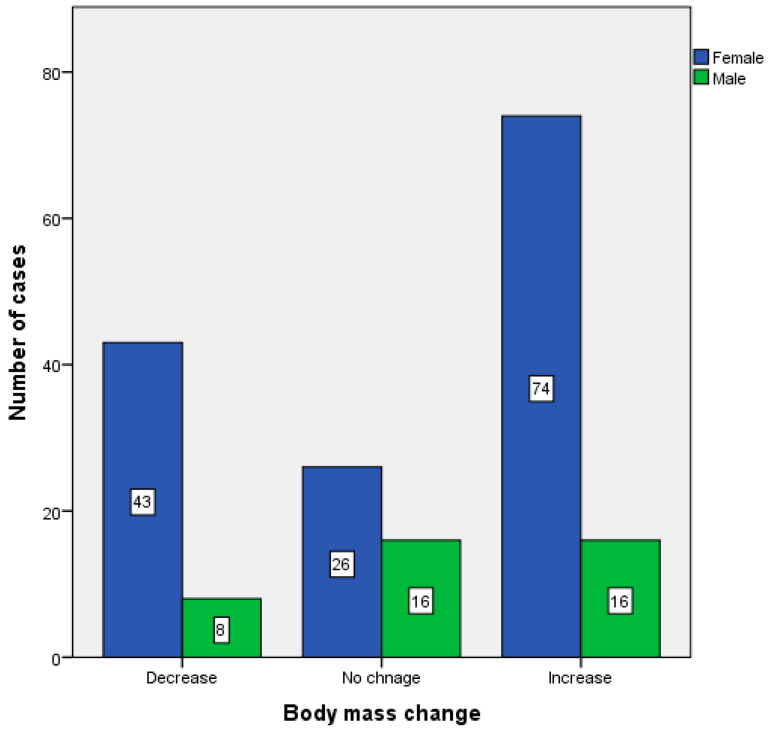
Change in body mass depending on sex in the study group during the lockdown.

Table 1 shows the results for the age, height, weight, and energy needs of the study participants. The mean age of the study participants was 33 ± 11, mean height 169 ± 8 cm. The body weight before the start of the lockdown was 69.8 ± 17.7 kg, while during the lockdown it increased to 70.4 ± 17.6 kg which shows an average increase in body weight by 0.63 ± 3.7 kg. The median change in body weight was 0 kg. The physical activity level decreased from 1.64 ± 0.15 to 1.58 ± 0.13. The above parameters show that before the start of the lockdown, BMR and TEE were respectively 1539 ± 250 kcal and 2532 ± 461 kcal and, during the lockdown, were 1546 ± 253 kcal and 2442 ± 429 kcal which constituted an increase in BMR by 6.99 ± 40.4 kcal and a decrease in TEE by 89.6 ± 234 kcal. All changes were statistically significant (*p* < 0.05, Wilcoxon test). Figure 1 shows the occurrence of changes in body weight depending on sex (Figure 1). Less than 30% (27.87%) of the study group reduced body weight during the lockdown. Among the study participants who did not lose weight, there was an average weight gain of 2.25 ± 2.5 kg (median body mass change 2.0 kg). Almost half (49.18%) of the study group increased their body weight during the lockdown. Table 2 shows the changes in body weight and energy needs of people who gained weight during the lockdown.

According to 20.2% of the respondents (*n* = 37), changes in body weight did not occur. Other study participants reported that the most common causes of changes in body weight were decreased physical activity (35%; *n* = 64) and the change of diet (20.2%; *n* = 37). Subsequently, there were changes in professional life (*n* = 18; 9.8%) and psychological factors (*n* = 14; 7.7%). Three participants (1.6%) indicated that the change in body weight occurred as a result of a food availability change, and 10 participants (5.5%) indicated other factors. The vast majority of study participants (*n* = 121; 66.1%) indicated no changes in the performance of heavy household duties.

**Figure 2:**
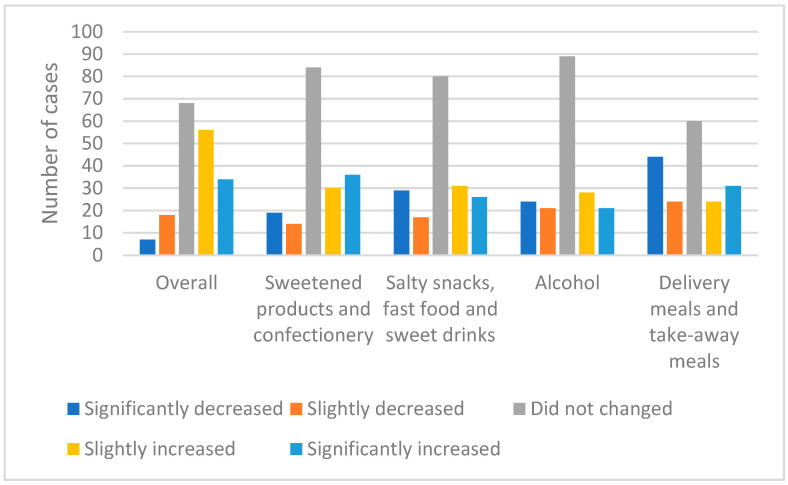
Changes in the structure of consumption of selected groups of products and food in general in respondents’ opinions.

Figure 2 shows the changes in total food consumption and the consumption structure of selected product groups in the studied group. Most often, the respondents declared no change in the total amount of food consumed as well as in individual product groups. In the case of total food consumption, an increase in consumption was declared by 48.4% of the surveyed group, while a decrease by 13.9%. The increase in the consumption of sweetened and confectionery products was observed by 36.2%, while a decrease by 18.6% of the respondents. Fast food and salty snacks were consumed in greater amounts by 32.4% of people. A total of 25% of the surveyed group declared lower consumption of these products. More alcohol was consumed by 26.6% of the respondents, and less by 24.5%. Further, 37.8% of the surveyed group used home delivery and take-away offers in restaurants less frequently, while 29.8% used these solutions more often.

It was shown that the change in body weight during the first lockdown was inversely related to the change in PAL (*p* < 0.001, rho = −0.324, Spearman test) and positively with the change in total food consumption, confectionery, fast food and take-away products (*p* < 0.001, Spearman test, for all groups, respectively: rho = 0.539; 0.601; 0.579; 0.281). An inverse correlation was found between the change in the rate of physical activity and age (*p* = 0.027, rho = 0.163, Spearman test), between the age of the respondents, a decrease in total food consumption (*p* = 0.02, rho = −0.171, Spearman test), and a positive correlation with an increase in the consumption of alcoholic beverages (*p* = 0.019, rho = 0.174, Spearman test). No other significant relationships were observed (*p* > 0.05, Spearman test).

## 4. Discussion

The COVID−19 pandemic has caused a number of changes in the lifestyle of people around the world, including Poland. Such drastic changes may increase the risk of adverse health consequences-obesity and chronic non-communicable diseases resulting from it.

The change in body weight in the entire study group during the first lockdown caused by the pandemic was relatively small and averaged about 0.6 kg, while the median change in body weight was 0 kg. It should be noted, however, that the follow-up period was very short. The first strict lockdown in Poland, which began in March 2020, lasted only half a month after which the restrictions began to be gradually loosened. Among other things, the ability to move around in public space was restored which could have resulted in increased activity in the study group and thus stopped mass gain. However, nearly half of the respondents gained weight. The change in body weight is a product of improper dietary energy intake compared to the daily energy needs of each study participant. Weight gain occurs when the energy balance is positive, i.e., the energy supply with the diet exceeds the body’s needs. As the analysis of the data obtained by us shows, almost 50% of the study group increased food consumption during the lockdown period and less than 14% decreased. It is interesting that a very similar number of study participants declared an increase in body weight and an increase in food consumption (49.2% vs. 48.4%) which was also shown as a strong correlation. In addition, it should be noted that people with a higher body weight often declared an increase in the consumption of products with high energy density, such as confectionery, sweets, salty snacks, and an increase in the consumption of take-away products, e.g., fast-food. Additionally, the decrease in physical activity contributed to the changes in body weight. A greater increase in body weight was observed with a decrease in physical activity (expressed as PAL). The vast majority of people taking part in the study changed their lifestyle to those commonly considered to be less healthy, excessive food consumption, increased consumption of energy-dense products, including largely low-nutritional products, increased alcohol consumption, and decreased daily physical activity. In addition, almost 70% of the respondents did not increase their energy expenditure by performing household chores that required increased physical activity. As a result, there was a positive energy balance because the total energy needs of the respondents decreased and the consumption of food, also with high energy density—increased. The small changes in body weight in the group are only the result of a short closure period. Longer-term restrictions on activity in the public space will only exacerbate this trend which may have serious consequences for public health in the long run. Obesity, in turn, increases the risk of developing many diseases, the presence of which often increased the risk of a more severe course of COVID-19 infection [16,17,18,19,20,21].

As in our study, the effect of reduced activity in public spaces caused by the COVID-19 pandemic on weight gain is reported by other authors. Pellegrini et al. (2020) reported an increase in body weight and a decrease in physical activity during the lockdown with increased total food consumption, as well as sweets and salty snacks [22]. Studies conducted in Lithuania, i.e., in a similar geographic location, showed a lower percentage of respondents who gained weight during the pandemic compared to this study, but also showed a reduction in physical activity and a comparable increase in total food consumption [23]. A significant decrease in physical activity and an increase in BMI were also observed in the studies conducted by Barrea et al. [24]. Studies conducted on a larger group in Poland also showed a decrease in physical activity [12], which may also lead to weight gain, especially as the results obtained in this study suggested an increase in food consumption in more than one third of the study group.

These observations in so many studies on often very numerous groups indicate that the results obtained by us are not unique. Leaving people locked up without proper support has led to negative changes in lifestyle and body mass. Only the short period of indoor confinement has limited the far-reaching consequences. The short period of closure in turn was due to the control of the epidemic situation in individual countries. A more infectious virus with a higher death rate would cause a much more restrictive form of lockdown over a longer period of time. This may lead into greater body mass gain which, without appropriate top-down intervention by national health authorities, would trigger an overall spread of obesity and related diseases.

It should be noted that not all respondents gained weight. Nearly 28% of the study group even reduced it. On the one hand, if it was not for these people, the average weight gain in the study group would be about 2 kg which underlines the seriousness of the situation. On the other hand, it means that a part of the study group was able to find motivation to maintain good eating habits and weight control by maintaining physical activity at home and controlling the quantity and quality of consumed food. There was almost no decrease in BMR in the studied group, while TEE decreased by less than 100 kcal. Thus, body mass control required only a slight reduction in the amount of food consumed. Further research on this group may provide information useful in the implementation of effective strategies to prevent overweight and obesity, as well as limiting the consumption of food products commonly considered unhealthy. It could be used to prepare intervention programs for the period of self-isolation and closure.

There are reports showing increased alcohol consumption in the period of social isolation and as a result of loneliness [25]. Moreover, studies conducted during the lockdown caused by the COVID-19 pandemic showed increased consumption of tobacco and alcohol compared to the period before the lockdown [26]. The increase in alcohol consumption shown in our study by more than a quarter, i.e., in a significant percentage of the study participants (26.6%), is a serious threat which should be given special attention. Alcohol abuse can lead to the development of addiction and health consequences, including the development of obesity and damage to many organs. The observed increase in alcohol consumption with age additionally increases the risk of complications caused by alcohol abuse, due to the involutional changes in the body that occur with age. Surely, attention should also be paid to the group that reduced the consumption of alcoholic beverages (24.5%), which is a positive observation, but the percentage of people who increased their consumption was higher which is still a significant problem.

Studies conducted by other authors similarly showed no changes in the consumption of alcohol in the vast majority of respondents [27]. Stanton et al. (2020) obtained similar results, where about half of the studied group did not change the amount of alcohol consumed, but as many as one fourth of this group increased its consumption [28]. Even more disturbing results were obtained on the basis of research conducted in Germany which showed an increase in alcohol consumption in more than one third of the respondents and no change in alcohol consumption in less than 40% [29]. However, controlling the amount of alcohol consumed may be crucial in preventing addiction and the negative consequences of excessive consumption of stimulants, especially alcohol. Numerous research observations indicate an increase in alcohol consumption during the lockdown which may have a negative impact on the health of the society.

Our research has some limitations. First, the questionnaire was conducted online which limits its accuracy. However, it was a safer form due to the persistent epidemic and the increase in the number of cases caused by SARS-CoV-2. Conducting the survey in this way protected the study group from possible infection. Secondly, a limitation is certainly the small size of the study group. It should also be emphasized that the study is retrospective in nature. The assessment of anthropometric parameters was based on the information obtained in the questionnaire, without verifying it with direct measurements. We did not measure body mass or height, or physical activity. Moreover, the assessment of changes in physical activity was made by means of a standardized questionnaire, not by measuring with direct methods. However, it should be emphasized again that conducting direct measurements in a group of people during an ongoing pandemic would increase the risk of COVID-19.

## 5. Conclusions

In conclusion, the study group has been shown to increase body weight in about half of the respondents. In the case of people who did not lose weight, the increase of about 2 kg occurred during a short limitation of activity in public space, as a result of the lockdown related to the COVID 19 pandemic at the beginning of 2020. Therefore, longer-lasting restrictions may lead to weight gain or excess body mass maintenance which, in turn, might be a risk factor for a more severe course of infection with the SARS-CoV-2. The observed increase in body weight was associated with a decrease in physical activity, as well as an increase in the consumption of total food and products with high energy density. Some of the respondents maintained or even decreased their body mass during the lockdown. Therefore, further research is needed, especially in people who have lost and/or retained body weight, to develop effective weight-loss prevention programs during periods of forced isolation by the pandemic.

## Figures and Tables

**Table 1:** Changes in body mass and energy needs in the study group during the lockdown (*n* = 183).

Phase	Age [Year] Mean ± SD Median Min-Max	Height [cm] Mean ± SD Median Min-Max	Weight [kg] Mean ± SD Median Min-Max	PAL Mean ± SD Median Min-Max	PPM [kcal] Mean ± SD Median Min-Max	CPM [kcal] Mean ± SD Median Min-Max
Before lockdown	33 ± 11 30 17–71	169 ± 8 168 150–195	69.8 ± 17.7 67 41–130	1.64 ± 0.15 1.6 1.6–2.2	1539 ± 250 1451 1082–2391	2532 ± 461 2441 1731–3825
After lockdown			70.4 ± 17.6 67 42–132	1.58 ± 0.13 1.5 1.5–2.2	1546 ± 253 1462 1082–2445	2442 ± 429 2338 1731 ± 3675
Significance *			*p* = 0.006	*p* < 0.001	*p* = 0.006	*p* < 0.001

* Wilcoxon test.

**Table 2:** Changes in body mass and energy needs of people who gained weight during the lockdown (*n* = 90).

Phase	Age [Year] Mean ± SD Median Min-Max	Height [cm] Mean ± SD Median Min-Max	Weight [kg] Mean ± SD Median Min-Max	PAL Mean ± SD Median Min-Max	PPM [kcal] Mean ± SD Median Min-Max	CPM [kcal] Mean ± SD Median Min-Max
Before lockdown	33 ± 11 30 17–63	170 ± 9 169 156–195	68.94 ± 16.5 66 41–130	1.65 ± 0.15 1.6 1.5–2.2	1526 ± 247 1435 1194–2391	2517 ± 458 2334 1791–3825
After lockdown			72.2 ± 17.5 69 42–132	1.54 ± 0.09 1.5 1.5–1.9	1561 ± 262 1466 1213–2445	2404 ± 399 2258 1820–3668
Significance *			*p* < 0.001	*p* < 0.001	*p* < 0.001	*p* < 0.001

* Wilcoxon test.

## Data Availability

The data presented in this study are available on request from the corresponding author. The data are not publicly available due to privacy of study participants.
